# A career in a Kingdom: Journeys in infection, Mass Gathering Medicine and public health diplomacy

**DOI:** 10.1080/21645515.2016.1226637

**Published:** 2016-08-25

**Authors:** Ziad Memish

**Affiliations:** Ministry of Health & College of Medicine, Alfaisal University, Riyadh, Kingdom of Saudi Arabia

A child of a diplomatic family, mine was a peripatetic childhood. My earliest memories are of traveling with my parents all around the world. Born in Mecca, I began my schooling immersed in diverse cultures: from Turkey to Jordan, from Oman to Saudi Arabia. The fifth among 6 siblings our family was mobile and dynamic because of my father who served the Kingdom of Saudi Arabia as a diplomat around the world. Because of him, my world was an ever-changing one—a world in which we traveled together as a family to wide and varying locations. Like every young child, in my eyes, my father was larger than life – and as a youngster I very much wanted to become a diplomat like him.

By the time of my higher education, I was coming of age in Canada. After two years of Pre-Med studies at King Abdulaziz University in Jeddah, Saudi Arabia, I was accepted into medical school at University of Ottawa, in Canada where my father was then posted.

Little did I know that while I had chosen an entirely different field of study to my father, subsequent turns in my career would lead to my own global journey, one in which I too would be tested as a diplomat in many capacities. 

Much of my work has focused on public health and even in this choice I see imprints of my childhood. As a very young child I noticed Saudi nationals seeking my father's help while he was stationed overseas. Of course, while it was his duty to serve our fellow nationals, the relief he brought in alleviating their distress had a lasting impact on me. I sensed he gained a special satisfaction from helping people in their time of need when they were far from the familiar and often far from family.

Forty years later, I have young children of my own, and in raising them I can see our family came of age in a different world. The Middle East was not nearly as connected internationally as it is now, but because our father's international appointments, I saw how the then young Kingdom served its peoples both inside the country and overseas.

No physician can claim authorship of his own career and I will never forget those who helped me sketch the outlines of my career path. Early in medical residency Professor Gary Garber the Chief of Infectious Diseases, Professor Bill Cameron a senior member of the Infectious Diseases division and Cathy Oxley, ART, CIC, the Director of the Infection Control program at Ottawa General Hospital, at the University of Ottawa invited me into the field through their intense curiosity, their devotion and their intellectual generosity: from *that* moment the die was cast—I was to be an infectious disease specialist too. It was *their* mentorship which ignited my passion for both infectious disease and infection control but, even more critically, instilled in me an appetite for academic enquiry and empiric research.

**Figure 1.**
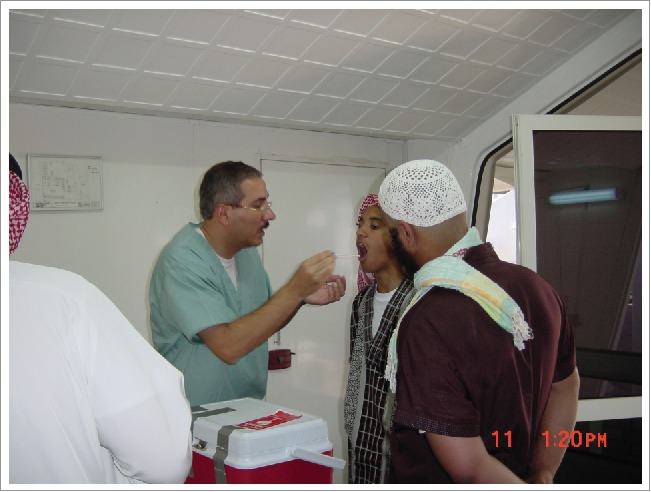
Ziad Memish at Mecca during Hajj 2008 taking throat swabs from a Muslim pilgrim.

Following the completion of my internal medicine and infectious diseases fellowships at the University of Ottawa, I was eager to sub-specialize in Infection Control. This was the most defining year in my training, during which I immersed myself into the field and took on a dozen infection control research projects. All the projects were presented at national and international meetings in addition to publication as original articles in peer reviewed journals. In hindsight, it is clear, that I was bitten by the ‘public health bug’, so to speak: gathering empiric data, analyzing it, and sharing exciting findings to advance knowledge had become my core value. I had found my professional calling.

Certainly there were times I despaired, just as any young physician adjusting to the demands of residency. Yet I was fortunate in my mentors who could see beyond my challenges at times when I could not. A particular night on call comes to mind, much as many of you may have experienced. I was overwhelmed with the work and felt powerless in the face of my patients and their complicated diseases. Rumpled from the difficult night, I told my professor my troubles, worrying I wasn't cut out for the profession. My professor patiently listened and with his experience, reframed my fears into a manageable size. Seeing him believe in my abilities restored my confidence at a critical time—when I had thought I would not continue in medicine. Fortunately for me, my professor knew otherwise!

This good fortune, of having others believe in me at times when my confidence ebbed, I have enjoyed throughout my career. I have been blessed to have enjoyed the support of an array of remarkable mentors from my earliest steps. Continually finding advocates and leaders who could see beyond my immediate capabilities was unquestionably a critical factor in advancing many of my professional goals. In later years, the generous and sincere faith others had in me would help me realize goals for my field and my country far beyond my imagination.

Despite an offer of an academic position in Canada, I decided to come back to Saudi Arabia, full of enthusiasm and drive to serve my country. I began immediately, finding myself at the frontier of modern infectious disease and infection control in the Kingdom. Saudi Arabia has a diverse healthcare system and in my time I would serve in many national health care structures: the Ministry of Interior Security Forces hospital, the Ministry of National Guard health affairs and most recently in the Saudi Ministry of Health to name some.

The Security Forces Hospital was my first exposure to advanced healthcare in Saudi Arabia and instructive not only to me clinically but also administratively. I learned greatly about the organization of healthcare in Saudi Arabia. It was an exciting time to work in the Kingdom. A year later I was recruited to the Saudi Arabian National Guard Health Affairs (NGHA) hospital by its CEO, then HE Dr. Fahad Alabduljabar, where I worked from 1994–2009. A flagship military medical institute, it was a career defining opportunity. I remain indebted to the NGHA leadership who gave me full and free rein to develop my practice, and pursue my academic enquiries through clinical research all the while supporting and developing my skills as an administrator. Our nation owes a debt of gratitude to the NGHA too—it had a huge and critical impact on the Kingdom at a time of intense development of its nascent public health system, and through a series of public health challenges. Consistently the NGHA leadership supported me while I managed first a division of infectious disease and infection control and later an entire department of Infection Control and Occupational Health. At this institute, which employed fifty-three different nationalities I was involved in helping engage and retain academic and clinical talent as we competed in a global market for the best minds and clinicians around the world. Our clinical resources expanded rapidly as we caught up with the first world international healthcare environment, doing so in mere decades. At this stage the NGHA became a well-recognized international institute and site for the highest tier of international research initiatives.

For these reasons, my personal affection for my colleagues and patients at the NGHA abides even today and the NGHA remains an important influence on me even today. There, I could pursue my areas of enquiry without restriction. My areas of interest were themselves a multidimensional journey, always led by the needs of Saudi Arabian's people.

Beginning with Brucellosis, an infectious disease which impacts many people in the Saudi Kingdom, my efforts were supported by the NGHA to communicate and collaborate with the Ministry of Health and the Ministry of Agriculture. Together we worked to reinforce animal vaccination strategies and devise an implementable but culturally acceptable public health education program discouraging the ingestion of raw camel's milk- widely regarded as a beneficial tribal practice. In these and many subsequent efforts the National Guard Health Affairs leadership's unwavering support fueled my strength and perseverance in making the institution an international beacon of excellence in Infection Control. Around this time, I established the Kingdom's first regional and international hub for Infection Control. Knowing how pivotal this was to public health, I personally lobbied for the recognition of Infection Control nationally in the Saudi Ministry of Health, and soon after, regionally, in the 6 Gulf Cooperation Council States (GCC). We in the Kingdom did this by developing a state of the art program in Infection Control as I envisioned it, inspired by my foundational mentors. With the help of talented and driven colleagues, our program became the reference standard, first for Saudi Arabia and, soon after, for the Middle East North Africa region.

By now, I was a member of a WHO Patient Safety committee in charge of developing global best hand hygiene practices and a member of an international Infection Control network encompassing Infection Control leaders from 6 WHO regions. Quickly, I was able to introduce the use of Alcohol Based Hand Rub as standard clinical practice in all healthcare facilities with the full support of Saudi Arabia's religious clergy despite the fact that alcohol is considered prohibited in Islam. The Saudi Arabian clergy confirmed what we as Muslim clinicians know- that only ingestion for intoxication is prohibited in Islam, and that medicinally alcohol is permissible.

Our experience engaging Saudi Arabia's clergy to teach Saudi physicians and patients about benefits of such products, eventually informed a novel disenchantment with alcohol hand rub developing in Western European Muslim population. The Kingdom's sophisticated experience of advancing public health through the nexus of medicine and religion lent critical insight to global hand hygiene campaigns and spearheaded efforts to find alternative, non-alcohol containing agents. These and other achievements in the field of Infection Control were recognized by the WHO leading to the designation of the NGHA as a WHO- and GCC States- Collaborating Center on Infection Control—a first for the Middle East North Africa Region. There we developed unified national and regional policies for Infection control which went on to be approved by the Ministers of Health of every GCC nation and quickly implemented into their own health care systems. Among my proudest achievements, with the help of the gifted team I assembled, the Kingdom was changing the health—for the better—of millions across the region.

Because of my early experience of wonderful and generous mentorship, the training of medical students, young physicians and nurses of all disciplines has long been my particular passion. Through the WHO Collaborating Center for Infection Control my membership on the first Board of Internal Medicine at the Saudi Commission for Health Specialties and my Associate Deanship College of Public Health and Health Informatics at King Saud bin Abdulaziz University for Health Sciences, I was proud to be involved with starting the first school of Public health in Saudi Arabia. I very much wanted future Saudi clinicians to experience the kind of academic environment I had had all those years ago in Canada, this time, right here at home. To facilitate publication of national and regional research in internationally recognized peer reviewed journal, I established the journal of infection and public health, published by Elsevier and with the support of an outstanding editorial team we were able to get it indexed in National Library of Medicine (NLM) in 2 y.

As I pushed for the Kingdom's first fellowship training programs in Infectious Diseases, I became the first Chair of the Board of Infectious Diseases training at the Saudi Commission for Health Specialties. It was not enough for me that our young doctors were to be trained locally, but that I also wanted them to meet the highest international standards—I knew they were capable and deserving of no less. My experience trailblazing training in Infectious Disease helped me develop the same for the field of Infection Control. In 2008 the Custodian of the Two Holy Mosques honored me with the King Abdulaziz Medal from the first degree. One of the highest national recognition for my service in the field of infectious diseases, infection control and public health.

While my colleagues have been generous in their assessment of my contributions (see Lancet 2014; 383(9934):e21; doi 10.1016/S0140-6736(14)60851-5), I know that there was much I had hoped both for myself and for my country to achieve. Perhaps the most instructive and challenging moment in my career was when I was called to serve the Kingdom as Deputy Minister of Health.

In this role I now had to think in entirely different dimensions. Moving to the Ministry of Health expanded my reach from one that was hospital- and academy-based to refocus on national and international policy making. I became the Assistant Deputy Minister for Preventive Medicine in 2009, assuming this position when the body responsible for governing public health within Saudi Arabia was itself in flux. Initially, the Department of Preventive Medicine was established within the Ministry of Health in response to the rising threat of Infectious diseases in the Kingdom and Arabian Peninsula. The department was tasked with preventing and controlling communicable diseases in the Kingdom. Under its guidance, incidence rates for many communicable diseases decreased significantly and Polio was eradicated in 1995. Since that time, the department has adapted to varied challenges and undergone a series of reorganizations and restructurings in response to the changing health needs and health status of the country's population.

As the socioeconomic status of the country has changed (urbanization, affluence, consumerism), an epidemiological shift has resulted in an alarming rise in the incidence and prevalence of non-communicable diseases (as in much of the first world) has now become the number one cause of death in Saudi Arabia causing 70% of all deaths. As such, the mandate of the Department of Preventive Medicine was expanded to focus on both communicable and non-communicable diseases.

In 2011, the Public Health Deputyship was restructured from the Department of Preventive Medicine in efforts to consolidate existing preventive programs while increasing their integration with Kingdom-wide primary care. Today the new Public Health Deputyship reports to the Deputy Minister for Public Health and, following my earlier efforts, I was appointed the first Deputy Minister for Public Health in Saudi Arabia.

It's clear that the Public Health Deputyship will become the central organizational and delivery body focused on improving the health status of the Saudi population. With the challenges we face in the Kingdom at the moment, its success is critical to the wellbeing of generations of Saudis. Fortunately, with the same support and endorsement of His Excellency the Minister of Health Dr. Abdullah A Alrabeeah and 2 Vice Ministers HE Dr. Mansour Alhowasi and HE Dr. Mohamed Khoshaim at that time, the Kingdom is committed to advance the national public health agenda at the highest levels underlining national priority on health indices.

Soon the public health deputyship refocused the vision of the Ministry of Finance on the budgetary needs of the preventative programs, shifting an earlier focus purely on curative medicine—it's only through prevention that the Kingdom can reduce the need for all costly curative programs like open heart surgery and oncology units. Work was rapidly started on the development of the Saudi CDC (Center for Disease Control) and as always, the commitment of the apex of our country's leadership to the needs of Saudi people, no less than a royal decree was issued to approve our plans and secure the necessary budget for a state of the art national Public Health Agency with huge laboratories focusing on high-level investigative research capabilities. Our center will become the first in the MENA region to house a Biosafety Level 4 facility—I was honored to be appointed its first director.

Our efforts in public health for the Kingdom had expanded exponentially over a very short time frame, stretching manpower. To expedite the development of national human resources to manage and run the now 46 national public health programs under the deputyship (26 Communicable Diseases programs, 11 Non Communicable Disease programs, and 9 Primary Health Care programs) at the Ministry of Health.

Internationally several Memoranda of Understanding were developed by the Kingdom and signed with key internationally recognized public health universities. These partnerships include an agreement with Emory University, renowned in the field of public health, to train Saudi physicians and administrators to obtain Master degrees in Public Health. Among my proudest accomplishments today over 46 Saudi citizens have completed the program and are currently assuming leadership positions across the Kingdom ensuring our nation's ongoing commitment to Public Health.

Elsewhere, the School of Tropical Medicine and Hygiene in Liverpool signed a Memorandum of Understanding to both establish and run international research center for tropical diseases in Gizan, situated in the Northern region of the Kingdom, where we will develop Saudi researchers, many of them engaged in basic bench research in state of the art laboratories seeking Master degrees and PhDs in Tropical Disease under the auspices of the Saudi CDC.

Much remains to be done. We are still short of manpower but have recognized this deficiency early on. We worked with the Ministry of Finance to approve the budget needed to establish formal IC positions in every one of its 320 institutions. In keeping with my belief that standards must match international ideals, we worked very closely with the Joint Commission International in the United States to develop a diploma in Infection Control for Saudi nationals to be conducted in KSA. The new Infection Control diploma, certified by the JCI, was rapidly recognized by the Saudi Commission for Health Specialties and the Saudi civil bureau with a capacity to train 60 candidates each year. At least 2 classes have been trained and certified with the new diploma and are already filling the newly created posts across all 320 Ministry of Health facilities. Similarly, an agreement with the University of Washington (Institute for Health Metrics Evaluation) to conduct the first national non-communicable diseases survey in the country, and to repeat this annually over a 5 y period ensured our MoH interventions in Public Health could be measured from their outset.

Comprehensive public health is only as good as its surveillance and reporting systems. To establish a reliable database on diseases in KSA, during my Public Health deputyship, we procured a state of the art electronic surveillance system and a national surveillance network called Health Electronic Surveillance Network (HESN) from IBM. We had implemented both systems Kingdom-wide within a 2 years' period- for the first time real-time, electronic surveillance of both Communicable and non-Communicable disease in Saudi Arabia had become possible. While the systems were a major cost burden, in one swift move we had eliminated paper based reporting through faxes to both an electronic notification system and alert system. Many first world countries are yet to acquire such sophisticated and comprehensive systems—in part because of the devotion of the monarchy to managing public health needs but also because the Kingdom, unlike many countries, is relatively unburdened with obstructive regulatory climates. To start documenting the achievements of the Saudi MoH, I established the second peer reviewed journal: Journal of Epidemiology and Global Health, a state of the art journal in the field of public health published by Elsevier and indexed in NLM in less than 2 y.

Soon after taking up the role as the Kingdom's public health steward, I was challenged with the emergence of H1N1 in 2009 which was quickly declared a pandemic by the World Health Organization. Our worst fears were realized as the pandemic coincided with the 2009 Hajj season.

To anyone who has not been to Hajj it is almost impossible to convey either the scale of the event or the spiritual gravitas to the world's largest mass gathering. An annual gathering of over 3 million pilgrims from over 180 countries, throughout the decades I have supported the Hajj season whether as a clinician, an academician, and more recently as a public health administrator. Without doubt, Hajj season continually challenged my resilience, my imagination, my physical and mental stamina and particularly my management skills. In 2009, for the first time in history, Hajj, a mass gathering, was being held during a declared global pandemic. Yet I never lose my sense of wonder and amazement at this remarkable rite which has continued uninterrupted for 15 centuries. Mecca is in a region of Saudi Arabia known as the Hijaz which has been a global gateway for millennia. Its people are accustomed to migrants, diverse populations and an ever-changing humanity. Beyond its mystique and magnetism, I am doubly blessed that Mecca would become the fulcrum to much of my career and ultimately ignite in me a desire to develop the field of Mass Gathering Medicine.

Challenged by the H1N1 pandemic, the Kingdom rapidly rose to the challenge as Hajj inevitably elevated KSA's prominence in the field of global health security. Our intense efforts during Hajj 2009 significantly strengthened the WHO International Health Regulations in 180 member countries participating in the Hajj.

With our already mature international networks and widely recognized public health systems, the Kingdom quickly galvanized support from global public health entities including the WHO and the US Centers for Disease Control and Prevention (CDC). With their collaboration, we mounted a strong, rapid and resilient response to the 2009 pH1N1 crisis—despite the millions of attendees from over 180 nations, only 5 pH1N1 deaths befell the event. Knowing more important even than the outcome was the knowledge we had gained in process, we rapidly identifying opportunities to share our experience with the global health community: this could only be done through the institutionalization of the discipline I saw to be realized in Mass Gathering Medicine. Working for so long at the frontier of Hajj medicine, I and other core pioneers understood an informal field was already coalescing as we collaborated in the management of large transient populations and the public health risks they entail but it was my personal vision and ambition that I ensured our nation's MoH Public Health Deputyship be designated as a WHO Collaborating Center on Mass Gatherings Medicine (in September 2012) and a global Center for Mass Gathering Medicine. Seeing the Kingdom realize this global first-establishment in the field of Mass Gathering Medicine, following Mecca's 15 unbroken centuries of hosting Hajj remains an achievement beyond my imagination.

Designations alone were not enough to sustain the Center and develop local capacities Incredible efforts and resources were mobilized to establish the first diploma in Mass Gathering medicine in KSA accredited by the Saudi Commission for Health Specialties.

Our efforts would prove invaluable only a short time later when the Kingdom faced a new emergent pathogen. ‘Mass Gatherings’ became one of the most significant global health security agenda items and the emergence of a new Corona virus: MERS-CoV. In Sept 2012, mere weeks before 2012 hajj season was to begin, this unknown agent put intense demands on both the Public health Deputyship and my entire team. While we had gained from the experience of H1N1 in our public health approaches and particularly the engagement of the international community, our newly formed MoH-international links and collaborations were put to the test in trying to understand this mysterious new virus.

Despite that effort and the enormous investment put by my government to address this new threat, I found more than a pathogen endangered the Kingdom and millions of ‘Guests of God’.

A seasoned public health diplomat long comfortable in international environments and critical negotiations, I was unprepared for my next challenge—I simply hadn't imagined it. In the fullness of the MERS crisis, for the first time I recognized certain international entities, rather than seeking collaboration in an effort to secure a vulnerable and massive population were baldly engaging in opportunist competition. This was a painful shock to me personally. I grew with wisdom as a result and I am proud of the work my colleagues are pursuing in this area today.

For now, I am returned to clinical and academic practice, taking a reprieve from public health diplomacy at this time. I enjoy teaching students at Princess Nora University, the world's largest university for women, and the Alfaisal University in Riyadh. At the bedside and in the clinic I reconnect with my origins: clinical infectious disease, infection control, academia and ongoing empiric research: the 4 pillars of practice I love most. If I have any regrets, it would be for the sacrifices my greatest love—my family—has made on my behalf. My intense work, and the challenging demands of a rapidly developing public health arena took me all too often away from my loved ones—my wife and my children. For this enormous sacrifice I continually ask for their forgiveness and in their unending generosities, somehow I continue to receive their unconditional love.

When I consider this extraordinary journey that one career in this Kingdom has bestowed upon me, possibly only one other experience gives me even greater solace than all the pandemics averted, all the Hajj seasons secured from disease and health hazards—that would be the mentoring of the young Saudi men and women rising in our field. I have taken singular pride in the achievements of my colleagues, many of whom have assumed the international stage alongside me. I salute these pioneering Saudi men and women as critical role models for our nation's future and important ambassadors of Islam's true spirit. We are proud of you.

I am now the father of three. My young children wait impatiently for me to finish this paper. Tonight as I look at my children, I think of the worlds they will confront, the increasingly globalized world they will move within and the novel challenges they may face. I can only imagine the interests and passions my young children will grow up to pursue but feel hopeful about the future as Saudi Arabia moves to a post petrochemical economy with a first world health system and a world class public health infrastructure, my children will be safe and healthy to explore, like their father once did, the fascinating world before them.

